# Robust and low-cost open-source device for detecting infectious microorganisms by loop-mediated isothermal amplification

**DOI:** 10.1016/j.ohx.2024.e00568

**Published:** 2024-08-24

**Authors:** Jorge Otero, Miguel A. Rodríguez-Lázaro, Arturo Martínez-Trejo, Daniel Mbanze, Gorka Solana, Andrea Vergara, Salvador Bosch, David Gozal, Jordi Vila, Ramon Farré

**Affiliations:** a Unit of Biophysics and Bioengineering, School of Medicine and Health Sciences, University of Barcelona. Casanova 143, 08036 Barcelona, Spain; b CIBER de Enfermedades Respiratorias. Monforte de Lemos 3-5, 28029 Madrid, Spain; c ISGlobal, Barcelona. Roselló 132, 08028 Barcelona, Spain; d Faculdade de Engenharias e Tecnologias, Universidade Save. Av. Américo Boavida s/n, Maxixe, Inhambane, Mozambique; e Hospital Clínic of Barcelona, University of Barcelona. Villaroel 170, 08036 Barcelona, Spain; f CIBER de Enfermedades Infecciosas. Monforte de Lemos 3-5, 28029 Madrid, Spain; g Department of Applied Physics, School of Physics, University of Barcelona. Martí Franqués 1, 08028 Barcelona, Spain; h Office of the Dean, Joan C. Edwards School of Medicine, Marshall University, 1600 Medical Center Dr, WV 25701, Huntington, WV, USA; i School of Medicine and Health Sciences, University of Barcelona. Casanova 143, 08036 Barcelona, Spain

**Keywords:** Infection detection, Low-cost medical device, Point-of-care diagnosis, Low-resources, bacterial/viral DNA

## Abstract

Loop-Mediated Isothermal Amplification (LAMP) is a useful technique for detecting infectious microorganisms in human fluids since it performs similarly to conventional PCR, the results are obtained faster and no thermocyclers or complex devices are required. Since only two isothermal blocks (95 °C to lyse cells and 65 °C for DNA amplification) are needed, LAMP is particularly suited for applications in Low- and Middle-Income Countries (LMICs). To validate such assumption, we first designed and tested Arduino-controlled LAMP thermoblocks to process a considerable number of samples simultaneously with a low-energy consumption to enable routine use under worst-case conditions (no main power source and low ambient temperatures). The thermoblocks were tested when battery-powered at temperature down to 5 °C, showing high stability in well temperatures (<0.8 °C). The charge required for both thermoblocks to simultaneously achieve the target temperatures after switching on and to keep their working temperatures were 4.1 A·h and 2.4 A·h/h, respectively. Second, we implemented a low-cost viewer with LEDs and filters to detect the fluorescent LAMP reaction. All the components required for the instrument are for general purpose and readily available by e-commerce. Thus, the LAMP device allows for considerable autonomy by using a typical car battery in rural and itinerant healthcare or field hospitals in LMICs, even under difficult environmental conditions.

## Graphical abstract

**Figure:**
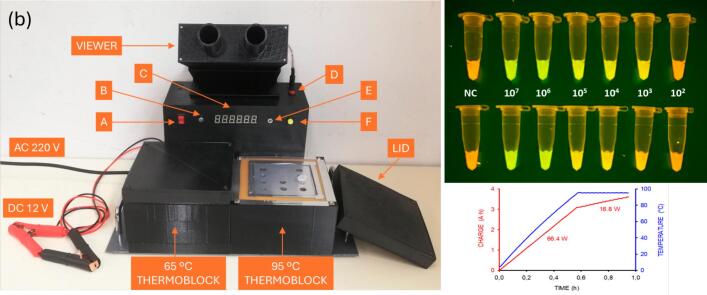

Specifications table

**Table:** 

Hardware name	Thermoblocks for Loop-Mediated Isothermal Amplification
Subject area	Medical
Hardware type	Biological sample handling and preparation
Closest commercial analog	No commercial analog is available
Open source license	GPL v3
Cost of hardware	The total cost of the material for the whole device building is ≈ 500 US$.
Source file repository	https://data.mendeley.com/datasets/8dnbrx3s6h/1

## 1 Hardware in context

The need for cheap and rapid tests to identify microorganisms causing infections is global and urgent. The ability to quickly detect the specific agents causing an infection would guide physicians in their choice of narrow-spectrum antimicrobial drugs rather than opt for wide-spectrum antibiotics, thereby preventing the emergence of antimicrobial-resistant strains while accelerating and improving therapeutic outcomes. For instance, pneumonia is a significant public health issue, being the 6th cause of death in the world [1] and the leading cause of child mortality from infectious diseases. Pneumonia leads to nearly 1 million estimated deaths annually [2], and primarily affects younger children (<5 years) in low and middle-income countries (LMICs). Microbiological diagnosis of the specific causative strain in these infectious diseases is critical to ensure appropriate selection of antibiotic therapy and to promote optimal outcomes along with decreased mortality [3].

Methods used to identify the specific microorganisms affecting any given patient, such as the Loop-Mediated Isothermal Amplification (LAMP) technique, are extremely useful when evaluating infectious diseases in health care settings [4]. LAMP is a relatively novel technique and offers potential advantages as compared to the conventional polymerase chain reaction (PCR) procedure. Indeed, it simplifies the readout of the results, as amplification can be visible by the naked eye directly in the LAMP reaction tube through a fluorescence assay or a colorimetric reaction (avoiding cross-contamination issues by opening the reaction tubes). Moreover, in contrast with PCR, the results are obtained more rapidly, and no thermocycling or complex devices are required. Since only two isothermal blocks (at 95 °C to lyse cells and at 65 °C for DNA amplification) are needed, identification of the infectious agent with LAMP can be implemented at a much lower cost than using PCR, which makes LAMP a particularly well-suited and attractive application in LMICs [4], [5].

Commercially available laboratory devices to carry out the isothermal processes at 65 °C and 95 °C are expensive, non-portable, and designed to operate at well-controlled laboratory temperatures while requiring stable power supply sources. Therefore, they are barely affordable and not well-suited for routine applications in most LMIC healthcare centers. To circumvent this problem, several low-cost devices have been proposed for LAMP [6], [7], [8], [9], [10], [11], [12], [13], [14], [15], [16], [17], most of them as recently as in the last 3–4 years. However, just a few of these devices have been distributed as open-source [6], [8], [9], [13], [16], [17]. Remarkably, even these devices have mainly focused on the 65 °C stage of the LAMP process, with very few including the previous critical step at 95 °C [9]. Moreover, most of them are just proof-of-concept prototypes for a reduced number of sample tubes with no robust construction. Finally, and possibly most importantly, these LAMP devices have not been evaluated for field use in challenging settings (e.g. unstable power supply) or shown their utility and reliability at extreme ambient temperatures. Therefore, the device presented here focuses on an important void since it addresses the limitations of extant applications while simulating the conditions usually found in LMICs, particularly in rural healthcare centers, itinerant medical services, or emergency field hospitals. Specifically, we describe an open-source, robust platform designed and tested to enable processing and visual identification of infectious organisms in a considerable number of LAMP samples simultaneously (10 at 95 °C and 32 at 65 °C) in conditions such as the absence of main AC power supply and at ambient temperatures down to 5 °C, below the regular range of use of molecular techniques devices in conventional air-conditioned stable ambient temperature clinical labs.

## 2 Hardware description

**Fig. 1:**
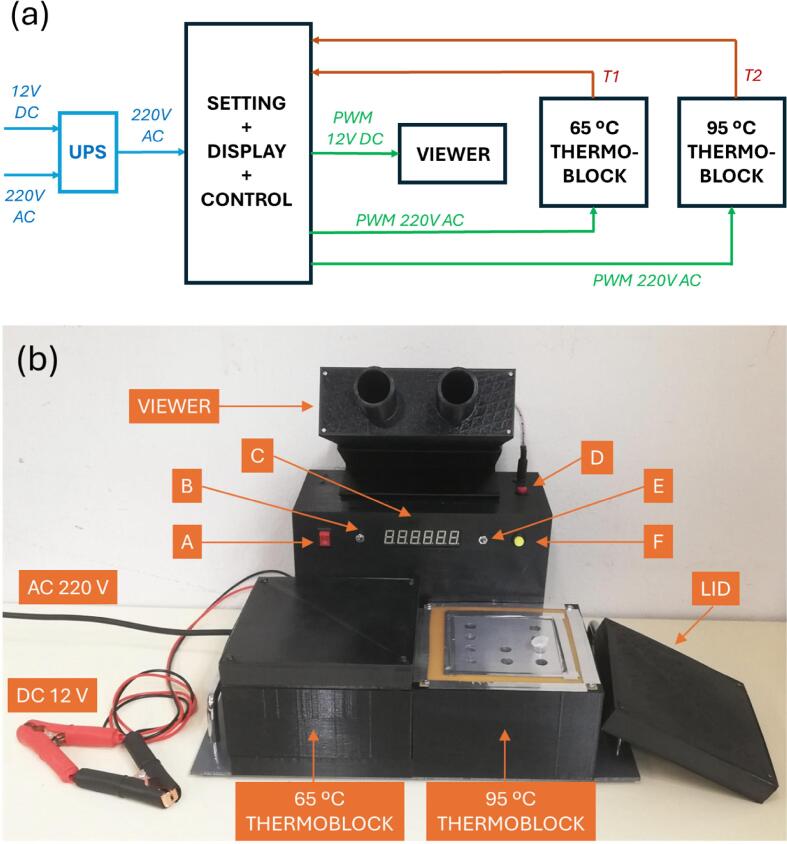
(a) Diagram of the LAMP device. The uninterrupted power system (UPS) component is represented in blue. The four main components of the device are represented in black: electronic unit (settings, display and Arduino-based control), thermoblocks for 65 °C and 95 °C, and the viewer.. (b) Picture of the built prototype with the lid of the 95 °C thermoblock open to show the wells for the Eppendorf tubes. A: general on/off button. B and E: on/off switches of the 65 °C and 95 °C thermoblocks respectively. C: temperature displays. D: button for lighting on the LEDs in the viewer and for setting their light intensity. F: button for establishing the temperatures settings.

An schematic description of the developed LAMP device is depicted in Fig. 1 a. A battery-based (compatible with a standard 12 V vehicle starter battery) uninterrupted power supply (UPS) system ensures that the device operates in the absence of AC mains power. The device has three main independent functions: two-thermoblocks to make possible the LAMP processes at both 95 °C and 65 °C in the biological samples, and a viewer to assess the results of the LAMP reaction visually. The device is operated by an electronic unit devoted to setting, displaying, and controlling the temperatures (T1 and T2) in the thermoblcks and the intensity of the LEDs inducing fluorescence in the viewer. A picture of the built prototype can be observed in Fig. 1 b.

### 2.1 Thermoblocks

The 95 °C (65 °C) thermoblocks consist of a 10x10x4 cm (10x10x2 cm) aluminum piece, with 10 (32) drilled wells on the top to allocate 1.5 mL (0.2 mL) Eppendorf tubes. Each block, incorporating a temperature sensor, is placed on top of a silicon heating pad (10x10 cm, 100 W, 220 V). The setting is thermally isolated with a 3-cm thick layer of mineral wool and contained in a 3D-printed enclosure including a lid. The temperature sensors signals are fed into the microcontroller (Arduino Nano) to regulate and to display them. The device is 220-V powered, either from a mains power or from a 12 V car battery and an inverter.

### 2.2 LAMP reaction detection

Given the typical relatively low intensity of the light emitted by LAMP samples as compared with normal ambient light intensity, the fluorescence results of the reaction have to be observed in the dark. As the availability of a dark room in certain application settings may be limited, the device has a viewer chamber to be used in any normally illuminated place. The viewer built in this specific implementation is aimed at detecting the LAMP reaction by the naked eye. The excitation light is produced by blue LEDs (470 nm) and a blue filter, and a secondary optical filter (blocking the excitation ligth wavelenght) is placed in the viewer.

## 3 Design files summary

All the design and software files necessary to build the LAMP device presented in this work (Table 1) are distributed under the GPL v3 license and they can be found in the supplementary materials of this manuscript at the following public repositories (https://doi.org/10.17632/8dnbrx3s6h.1):

**Table 1:** Files summary.

**Design file name**	**File type**	**Open source license**	**Location of the file**
Enclosures and lids	STL	GPL v3	STL files folder
Code	ino file	GPL v3	Arduino Code folder
PCB Layout	pdf and jpg file	GPL v3	Electronics folder

https://data.mendeley.com/datasets/8dnbrx3s6h/1.

## 4 Bill of materials summary

**Table:** 

**Component**	**Quantity**	**Cost per unity €**	**Total Cost currency €**	**Source of materials**
DC-AC SSR-80DD 80A 3-32VDC a 5–200 VDC	*1*	16,43	16,43	https://www.amazon.es/dp/B0BG34V7LR/ref = sspa_dk_detail_4?psc = 1&pd_rd_i = B0BG34V7LR&pd_rd_w = 6Kzs5&content-id = amzn1.sym.bc0d9e84-6f8a-453b-bbd2-622e2ccbd3a9&pf_rd_p = bc0d9e84-6f8a-453b-bbd2-622e2ccbd3a9&pf_rd_r = PB0VFD3W6SNM3FJVR5ND&pd_rd_wg = mWbvB&pd_rd_r = afb613b1-fda6-4a72-ab70-98901fcdc6ac&sp_csd = d2lkZ2V0TmFtZT1zcF9kZXRhaWxfdGhlbWF0aWM
Relay Finder 40.52.9.012 DPDT	*1*	7.03	7.03	https://www.amazon.es/Finder-serie-40-reticulado-conmutado/dp/B0018L3QJW/ref = sr_1_5?__mk_es_ES=%C3%85 M%C3%85 %C5%BD%C3%95 %C3%91&crid = 3DFRFUWE2DVKN&keywords = rele%2Bdoble&qid = 1707129187&s = industrial&sprefix = rele%2Bdoble%2Cindustrial%2C103&sr = 1–5&th = 1
SERADHE heater Dissipater 1138.3 70X64X25 TO-3	*1*	8,68	8,68	https://www.ondaradio.es/Catalogo-Detalle/1439/radiadores-seradhe-estrusion/20446/rohs-seradhe-radiador-11383-70x64x
Resistors 4.7KΩ,	*2*	0.013	0.026	Local store
Resistors 8.2KΩ,	*4*	0.013	0.052	Local store
Resistors 220 Ω,	*2*	0.013	0.026	Local store
Resistors 1MΩ, 0.5 W	*1*	0.013	0.013	Local store
Resistors 560 Ω,	*1*	0.013	0.013	Local store
Resistors 1.2KΩ,	*3*	0.013	0.013	Local store
LEDs	*18*	0.263	4.734	https://es.rs-online.com/web/p/leds/8100482?searchId = acab8f36-7368-4d7a-899f-987dfeb5d431&gb = s
Transistor 2 N2222	*2*	7.99	15.98	Local store
Diod 1 N4007	*4*	0.053	0.206	Local store
IC H11L2	*1*	0.4	0.4	Local store
Capacitor 100μF,25 V	*3*	0.8	2.4	Local store
Capacitor 220μF, 25 V	*1*	0.8	0.8	Local store
Capacitor 680NF, 400 V	*1*	5.21	5.21	Local store
Diod Zener 4.7 V, 1 W	*1*	0.035	0.35	Local store
220 V AC Connector	*1*	2.66	2.66	https://www.amazon.es/Chasis-Montar-Clavija-Conector-6-35mmTABS/dp/B003OSUIRK/ref = sr_1_9?dib = eyJ2IjoiMSJ9.sTLfXgHRKOJa4zVRNBz9Rylp2Yc_cqBAwjVmsK4ba1KIciPuXbxluO7F8nSyH5e-auwW88hYZRX8gGv6cZxaAaU_laZ35eI2s3X-wktFpqqLeK5MG9a7dbXGZE8eO-V5Lx3rY4Cjkpo65ajmV0EbJX7Y9D-mRkuTu2_MOsyPzTAtatNsZ1GDsiEzR2nGzgc0TScFyT6ZGXmkPo0aM7aOmb75OZ9rtKCW1Enaoe-1h2alrPuFQXXVby-HCZIRrrBpTj8IVtrJkfrchnEAGs3xKCHt-MLUsdX6CjBrsU8ZTVs.3IMJq8_YkrRcYMR8436uReGwt2xlZiCcPcK8lqYR9Y4&dib_tag = se&keywords = panel + iec + 320 + c14 + conector&qid = 1715252391&sr = 8–9
DC-022 DC Power Jack Plug 5.5x2.1 mm Connector	*1*	6.99	6.99	https://www.amazon.es/dp/B08218HN5H/ref = sspa_dk_detail_8?psc = 1&pd_rd_i = B08218HN5H&pd_rd_w = 04KRx&content-id = amzn1.sym.f4077e72-eba9-451d-9ae6-34c639219763&pf_rd_p = f4077e72-eba9-451d-9ae6-34c639219763&pf_rd_r = T28X7699K1TJSYC85M8J&pd_rd_wg = knxz5&pd_rd_r = 3ec35430-5958-42b7-86a6-7370251d476b&s = industrial&sp_csd = d2lkZ2V0TmFtZT1zcF9kZXRhaWxfdGhlbWF0aWM
IC7805	*1*	0.28	0.28	Local store
Connectors 1x2	*8*	0.021	0.168	Local store
Connectors 1x3,	*5*	0.021	0.105	Local store
Connectors 1x4,	*2*	0.021	0.042	Local store
PCB 60x80mm	*2*	0.65	1.3	Local store
2 pins AC 6A/250 V 10A/125 V 2 on/off Switch	*1*	10.50	10.50	https://www.amazon.es/dp/B07DYL7V83/ref = sspa_dk_detail_2?pf_rd_p = f4077e72-eba9-451d-9ae6-34c639219763&pf_rd_r = 1T5VHX000B9HQG19422N&pd_rd_wg = 3e0IY&pd_rd_w = TrRBg&content-id = amzn1.sym.f4077e72-eba9-451d-9ae6-34c639219763&pd_rd_r = 76345414-a1cb-448c-ab1a-d7c5b1d7b80c&s = automotive&sp_csd = d2lkZ2V0TmFtZT1zcF9kZXRhaWxfdGhlbWF0aWM&th = 1
2P 3A 250VAC/6A 125VAC ON/OFF Switch	*2*	11.09	22.18	https://www.amazon.es/Mintice-Interruptor-Basculante-Miniatura-Salpicadero/dp/B01K7E3FZA/ref = sr_1_27?__mk_es_ES=%C3%85 M%C3%85 %C5%BD%C3%95 %C3%91&crid = ILV8GGCDGJ9C&dib = eyJ2IjoiMSJ9.oUtxkbPgAX-MRAZ7FSDRLBmBA7NpIv9YT9Ij1kp3hZHGjHj071QN20LucGBJIEps.uFeKVUsxbK8wU4xZnT47asA38Tlm9Bq5MqxPhmoHMxo&dib_tag = se&keywords = interruptor%2Bpalanca%2Bpanel&qid = 1715252765&s = automotive&sprefix = interruptor%2Bpalanca%2Bpanel%2Cautomotive%2C89&sr = 1–27&th = 1
Button Switch 3A 125 V AC/1,5A 250 V AC	*2*	0.36	0.72	https://www.amazon.es/interruptor-pulsador-12 mm%EF%BC%8CInterruptor-Bloqueo-redondos/dp/B07T1ZLLPW/ref = sr_1_13_sspa?__mk_es_ES=%C3%85 M%C3%85 %C5%BD%C3%95 %C3%91&crid = 2UNGAJEFA7IVY&dib = eyJ2IjoiMSJ9.vBTgWOlR3UQKjYmB8cAxPJrn3D6W4aB3jnaUdGqq6GAw2gE3d753NbOg5eFzkCr4-fF5zfnD36ACxcLCCWVyusRtlKG1fnAgnhoIkma7vP16CkGGfMuZVdo5z0lhJjxRwvt3uXrzCwl_gr2w7evVkH9zYD1JPa6YANA4Y_YR7uL1OERyfSbae2F9L7f-6FVSk6U9v2Ks-UcPDOqgjPD0lFHhsiNW6vksEMoYtpKHADcVeffYOydzS7-QxghNiOkbX64YF_2j8RdHC6QaK7lbR1OhNodfaMM4dQYF6XOZguw.Rd-euAexnrgGqTILIec7wkS0Id5MZz89u7Sm-7YXWHQ&dib_tag = se&keywords = pulsador%2Bpanel&qid = 1715252882&s = automotive&sprefix = pulsador%2Bpanel%2Cautomotive%2C88&sr = 1–13-spons&sp_csd = d2lkZ2V0TmFtZT1zcF9tdGY&th = 1
Arduino Nano	*1*	6	6	https://www.amazon.es/ELEGOO-ATmega328P-Compatible-Arduino-Proyecto/dp/B0716T2L77
500 W Power InverterInput 12 V DC,Output 210–240 V AC,USB: DC 5 V 3.5A.	1	41.99	41.99	https://www.amazon.es/Yinleader-Corriente-220 V-240 V-convertidor-Transformador/dp/B091GCQ566/ref = cm_cr_arp_d_product_top?ie = UTF8
Cable fusible 30A	1	1.4	1.4	https://www.amazon.es/Gebildet-Portafusibles-Impermeable-Extractor-Fusibles/dp/B07XR9BBK4/ref = sr_1_3_sspa?__mk_es_ES=%C3%85 M%C3%85 %C5%BD%C3%95 %C3%91&crid = 27WYVDSPV1V8I&dib = eyJ2IjoiMSJ9.WVVBPns-KbeyZspX68saqrY1BjFM9Bcn181RRgSCplNXAMziTuY3KfF3QDZJjMasgYE_-RdyHeGlIfwoMooeZrYaeY8OpL3bvsuXwFWGS4o8b0lvnvyeKsESNFyQNOc8jqgcA3HvqiKMo4wzPz5DG-pInSMN38TTLKMcmlY7HWHpJsOU0t93xgLidOcrscu1T8BhNeEA7rK7WF20nJQ5s5e0athptWHaCjESGD3o3e5_W6sT0l9EdzNZBCAOjLG089lijEMg8WZX7vuD4EI7NGvYMlQGXqrU5jktmmW0EGM.v4GHLbMyoI2LjdpvR0F1ukZOySsbaOjQugNZKZC-sHg&dib_tag = se&keywords = Cable + fusible + 30A&qid = 1715255936&sprefix = cable + fusible + 30a%2Caps%2C100&sr = 8–3-spons&sp_csd = d2lkZ2V0TmFtZT1zcF9hdGY&psc = 1
Battery charger cables 30A, 1 m longitudinal	1	9.99	9.99	https://www.amazon.es/Greluma-cocodrilo-Cargador-bater%C3%ADa-autom%C3%B3vil-1 m/dp/B09TLDX5QG/ref = sr_1_1_sspa?__mk_es_ES=%C3%85 M%C3%85 %C5%BD%C3%95 %C3%91&crid = W6GBI9JZS85J&dib = eyJ2IjoiMSJ9.MSIG22ibz2z9sEbArxWVRokcvjAhpjInZ0uD9h8H5bJGnwlN-FnorC17q9fz4S4ojjA38u0hbkS5YmdVLG7bCHWuilE5kKZg_nrDS4HSXEl-BxzbcwtDv8T6Xa0uc01VKrgOdw_g07ViM28kHxj-thSV32AIY_OsQSZuxQVqIW_7ySGXPKbmjQR3_lVZ9e0r9ZXgcUQ1Sj7oIhHCVDh1FMwJPvBsL2OFYg3DmP2v3deYGFRm-5nvds4bZjlz1lNGN--I8sEeDtY0uuNP4TjD0w_JE5H44JkGRyb6Pjte69s.xoSJc2lRSlxQX9Jj2BEolhk3trIjlHUnLh1j9FBJ_vE&dib_tag = se&keywords = Piezas + 30A+Pinzas + de + cocodrilo + 1 + metro + longitud&qid = 1708114241&sprefix = piezas + 30a + pinzas + de + cocodrilo + 1 + metro + longitud%2Caps%2C196&sr = 8–1-spons&sp_csd = d2lkZ2V0TmFtZT1zcF9hdGY&psc = 1
Terminal junction m6 poster red and black	2	1.83	3.66	https://www.amazon.es/QWORK-Terminal-encuadernaci%C3%B3n-terminales-Conector/dp/B09GFP162P/ref = pd_rhf_cr_s_pd_crcd_d_sccl_{214/258–7138868-7732429?pd_rd_w = NU9q5&content-id = amzn1.sym.09801950-5e05-490a-a4a2-44fc5919454c&pf_rd_p = 09801950-5e05-490a-a4a2-44fc5919454c&pf_rd_r = M45P7MJKFW4SVFR28XSS&pd_rd_wg = q8nOb&pd_rd_r = d47cfc02-c6fe-42cb-851c-e8b62cccce25&pd_rd_i = B09GFLSD43&th = 1https://https://www.amazon.es/dp/B09TLDX5QG/ref = sspa_dk_detail_5
DS18B20 Digital Temperature Sensor	2	7,99	15,98	https://www.amazon.es/dp/B01MZG48OE/ref = sspa_dk_detail_2?pf_rd_p = f4077e72-eba9-451d-9ae6-34c639219763&pf_rd_r = JTVKEN0QMG0GC6YWHYZ3&pd_rd_wg = i8PEx&pd_rd_w = LqLUs&content-id = amzn1.sym.f4077e72-eba9-451d-9ae6-34c639219763&pd_rd_r = 0f7aeba7-8199-43b9-a685-cbc2274d4666&s = industrial&sp_csd = d2lkZ2V0TmFtZT1zcF9kZXRhaWxfdGhlbWF0aWM&th = 1
DC voltage source 12 V	1	8.19	8.19	https://www.amazon.es/gp/product/B09VDCRR89/ref = ox_sc_act_image_1?smid = A1T5PV1D2YX89Q&th = 1
Aluminum block 100x100x20mm	1	29.82	29.82	https://es.aliexpress.com/item/1005003473047506.html?src = google&src = google&albch = shopping&acnt = 439–079-4345&slnk=&plac=&mtctp=&albbt = Google_7_shopping&albagn = 888888&isSmbAutoCall = false&needSmbHouyi = false&albcp = 18928172568&albag=&trgt=&crea = es1005003473047506&netw = x&device = c&albpg=&albpd = es1005003473047506&gad_source = 1&gclid = CjwKCAiA3aeqBhBzEiwAxFiOBjobBEharGOUI48EbUsibJLDmRpYrPbZx9EfJFR371K0wdZUwGoewBoCmUkQAvD_BwE&gclsrc = aw.ds&aff_fcid = 74b23c3b64304dc0acccd903fe789026-1699348013115–05892-UneMJZVf&aff_fsk = UneMJZVf&aff_platform = aaf&sk = UneMJZVf&aff_trace_key = 74b23c3b64304dc0acccd903fe789026-1699348013115–05892-UneMJZVf&terminal_id = 237903a7cdcd47e399c99f702252f747&afSmartRedirect = y
Aluminum block 100x100x40 mm	1	53.45	53.45	https://es.aliexpress.com/item/1005003473047506.html?src = google&src = google&albch = shopping&acnt = 439–079-4345&slnk=&plac=&mtctp=&albbt = Google_7_shopping&albagn = 888888&isSmbAutoCall = false&needSmbHouyi = false&albcp = 18928172568&albag=&trgt=&crea = es1005003473047506&netw = x&device = c&albpg=&albpd = es1005003473047506&gad_source = 1&gclid = CjwKCAiA3aeqBhBzEiwAxFiOBjobBEharGOUI48EbUsibJLDmRpYrPbZx9EfJFR371K0wdZUwGoewBoCmUkQAvD_BwE&gclsrc = aw.ds&aff_fcid = 74b23c3b64304dc0acccd903fe789026-1699348013115–05892-UneMJZVf&aff_fsk = UneMJZVf&aff_platform = aaf&sk = UneMJZVf&aff_trace_key = 74b23c3b64304dc0acccd903fe789026-1699348013115–05892-UneMJZVf&terminal_id = 237903a7cdcd47e399c99f702252f747&afSmartRedirect = y
Silicon heating pad 100x100 mm, 100 W, 220 V AC	2	24.13	48.26	https://www.amazon.es/Zunate-Almohadilla-Impresora-Calefactora-Impresoras/dp/B0BFX4M49Z/ref = sr_1_24?__mk_es_ES=%C3%85 M%C3%85 %C5%BD%C3%95 %C3%91&crid = 1F1WA29H8M5JW&keywords = Almohadilla + calefactora + de + goma + de + silicona + 220v&qid = 1698655640&sprefix = almohadilla + calefactora + de + goma + de + silicona + 220v%2Caps%2C83&sr = 8–24
Fiberglass Plates C40695, 1.5 mm	2	14,60	29.2	https://www.amazon.es/dp/B07G8CTR63/ref = sspa_dk_detail_4?psc = 1&pd_rd_i = B07G8CTR63&pd_rd_w = 1w1V7&content-id = amzn1.sym.9c67f205-18e7-4d34-beb2-37ec708092ed&pf_rd_p = 9c67f205-18e7-4d34-beb2-37ec708092ed&pf_rd_r = 12GE11VTK1MGDR8WNFVB&pd_rd_wg = xWz8h&pd_rd_r = 241a61fb-2ced-4457-9b38-0423fe9a96cd&s = tools&sp_csd = d2lkZ2V0TmFtZT1zcF9kZXRhaWw
6 Digital Display LED TM1637, 0.56″	1	9.29	9.29	https://www.amazon.es/visualizaci%C3%B3n-segmentos-pulgadas-pantallas-optoelectr%C3%B3nicas/dp/B0B914MY5J/ref = pd_sbs_sccl_4_7/262–5718688-8116448?pd_rd_w = G5qeo&content-id = amzn1.sym.5e7265fb-282c-45b2-9c89-a01e01f0385b&pf_rd_p = 5e7265fb-282c-45b2-9c89-a01e01f0385b&pf_rd_r = 27KFA2VG3Q6H3B3TVTQS&pd_rd_wg = RZ1df&pd_rd_r = 436b2d69-8c69-4b2d-b921-d60925dad7ce&pd_rd_i = B0B914MY5J&psc = 1
Ultimaker PLA Filament 1Kg, 2.85 mm	2	40	80	https://es.rs-online.com/web/p/materiales-*para*-impresion-3d/1830259
Polycarbonate Filament 180 g, 2.85 mm	0.18	66.66	23.99	Local Store
HALJIA 2 canal 5 V modulo, solid stage relay	1	9.99	9,99	https://www.amazon.es/resistiva-Fusible-Duemilanove-MEGA2560-MEGA1280-ARM-Raspberry/dp/B06XF1TCR3/ref = sr_1_4?__mk_es_ES=%C3%85 M%C3%85 %C5%BD%C3%95 %C3%91&crid = 23KLIYID3TETV&keywords = rele + estado + solido + arduino&qid = 1698670022&s = industrial&sprefix = rele + estado + solido + arduino%2Cindustrial%2C90&sr = 1–4
LEDs (470-nm, C503B-BCS-CV0Z0462, Cree LED)	16	0.216	3.46	https://es.rs-online.com/web/p/leds/8100381
Blue filter, plastic screen (078-Tokyo Blue, LEE Filters)	1	5.30	5.30	https://www.thomann.de/es/lee_colour_filter_071_tokyo_blue.htm
Plastic screen (015-Deep Straw, LEE Filters)	1	5.30	5.30	https://www.thomann.de/es/lee_farbfolie_nr015_deep_straw.htm

The total cost of the materials for building the device, including its UPS component is 492.58 € (~500 US$). Materials such as resistors, LEDs, pin connectors, capacitors, PCB, ICs, and fuse holders were purchased as a kit, however, not all materials available in the set were used when building a single device. Most of them can be also easily reused from obsolete/damaged consumer electronic devices or household appliances.

## 5 Build instructions

### 5.1 3D design and printing

**Fig. 2:**
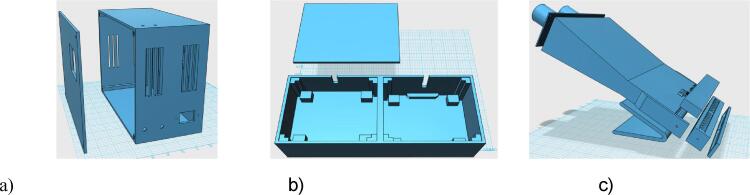
Enclosures for electronic devices (a), for the aluminum blocks (b), and for LED Photodiode Box (c).

Electronic and thermal circuits ware assembled in a 3D-printed enclosure including the lids and they are fixed with appropriate screws. The enclosures (Fig. 2, a and b) were designed to offer adequate mechanical resistance and they are printed in polycarbonate, material which is highly resistant to the thermal operating conditions of the thermoblocks. Designs and can be viewed and modified in the distributed STL files.

The LAMP viewer enclosure is 3D-printed too, and it is assembled as shown in Fig. 2 (c). The top is made up of a lid with two cylindrical holes through which the observer can approach his/her eyes. Its interior is hollow, allowing visibility between the top and bottom. At the bottom, a bar containing 16 holes is fixed for placing the samples. Each hole is equipped with an LED, that has an adjustable light intensity from 0 to 100 %(10 different levels of intensity). The lids are fixed on the bar to close the holes directing the light to provide an appopiate view to the observer to evaluate the results of the reaction.

**Fig. 3:**
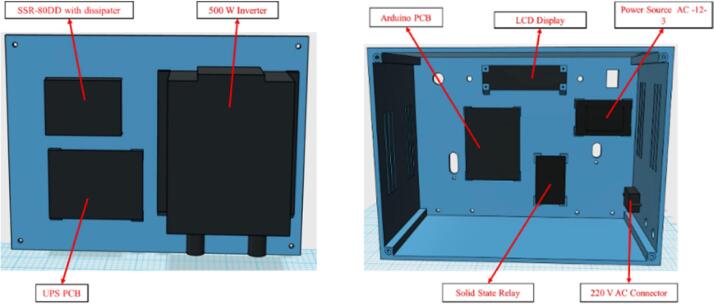
Electronic device assembling on the lid (left) and enclosure (right).

The electronic boards and other components are fixed and screwed inside the enclosure as well as on the lid, in the previously defined spaces according to their dimensions, as shown in Fig. 3.

**Fig. 4:**
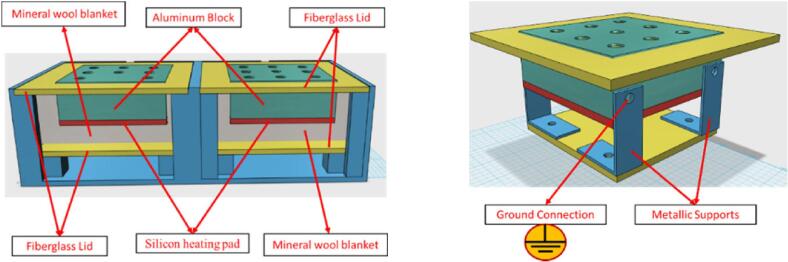
Thermoblocks assembling (left) and fixing the aluminum block and grounding (right).

The aluminum blocks are isolated from the enclosures by using fiberglass lids fixed with screws, including a mineral wool blanket that fills the inner part of the enclosures. The blocks are fixed to the lower fiberglass lid using screwed metallic supports as shown in Fig. 4. The aluminum blocks and supports are electrically grounded.

### 5.2 Electronics

**Fig. 5:**
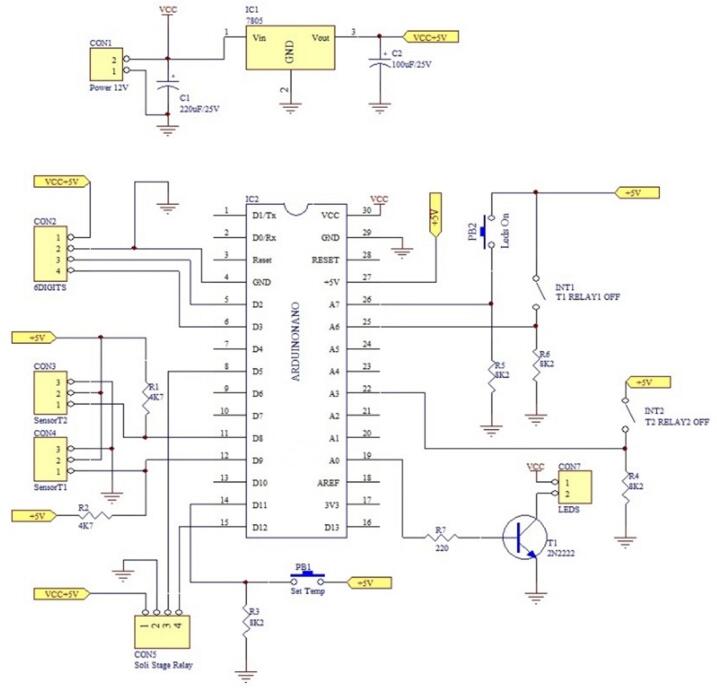
Schematic of the electronic circuit.

**Fig. 6:**
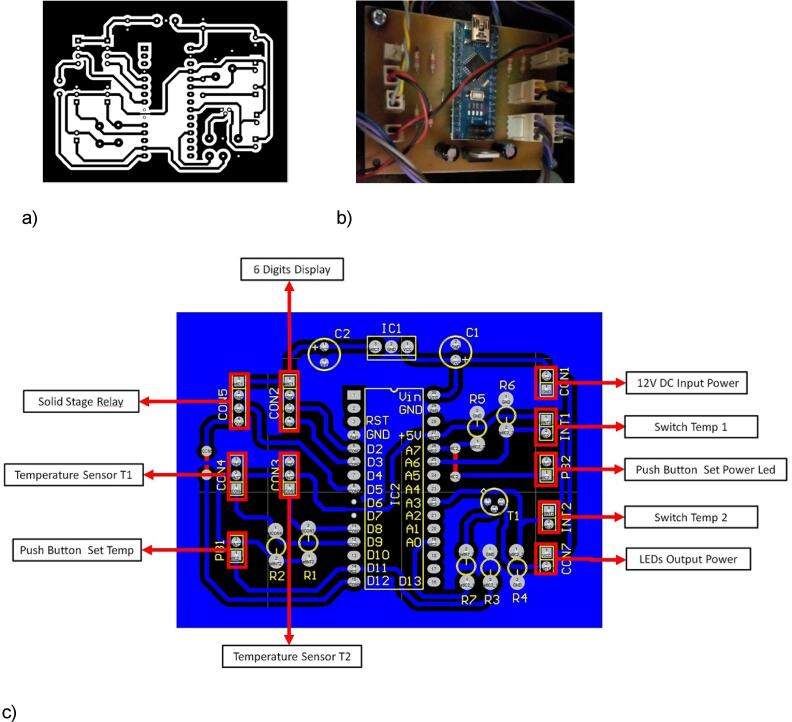
PCB board tracks (a), Soldered components (b), and connection pins(c).

The schematic of the implemented electronic circuit is shown in Fig. 5. The Printed Circuit Board (PCB) was designed using the PROTEL99SE software. The PCB layout of 5.83 cm × 8.09 cm can be envisioned in Fig. 6 as well as attached in JPG format in the PCB folder, either for the Arduino controller circuit as well as for the UPS circuit. The PCB is placed in the drawer by using screws. Then, the frontal wall of this structure is included: it presents the general power on/off, the 6-digit LCD screen, the LEDs, the Button for setting the thermoblock's target temperatures, and the on/off switch for each Thermoblocks. The heater and the DS18B20 digital temperature sensors are connected to their respective pins. Regarding the heater, thicker copper wires, with a minimum of 1.5 mm^2^ of cross-section are used to sustain the required intensities.

### 5.3 Leds circuit connection

**Fig. 7:**
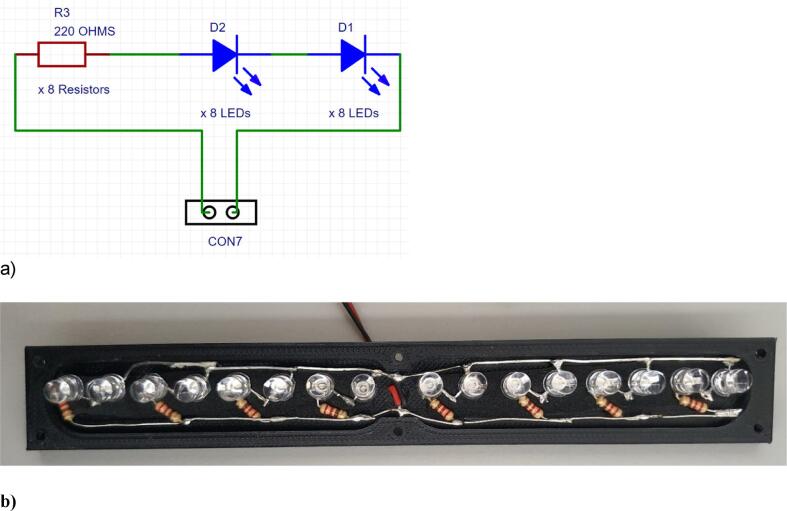
Branch LEDs Circuit Diagram **(a)** and Power LEDs Circuit Assembling **(b)**.

**Fig. 8:**
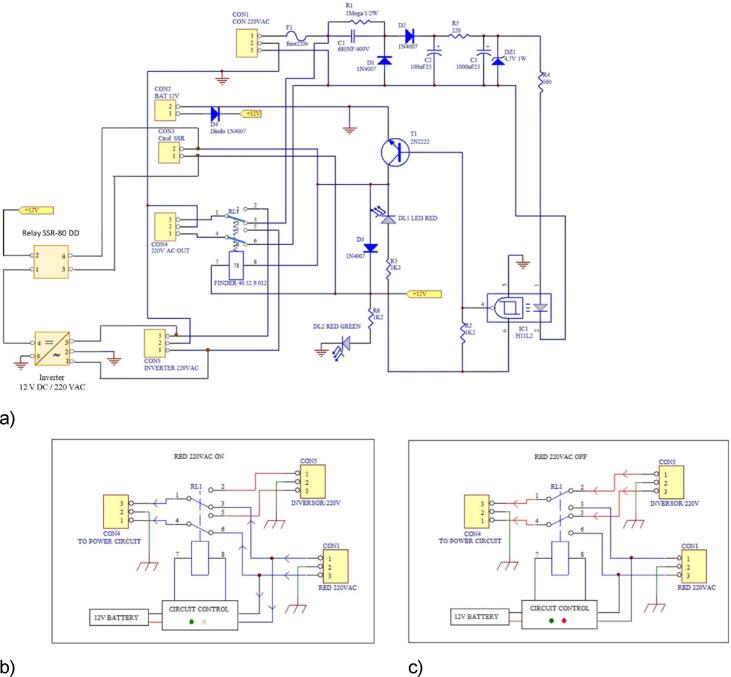
Schematic of the UPS electronic circuit (a), the operation when sourced from 220 VAC conventional electrical network (b), and operation when sourced from 12 VDC battery (c).

A total of 8 branches (powered at 12 VDC) are connected in parallel association in the built LED power circuit as can be seen in Fig. 7, b. Each branch comprises a serial association made of two LEDs and one 220 Ω resistor (see Fig. 7, a). The final assembly of the LED Circuit can be seen in Fig. 8 b).

### 5.4 UPS circuit design

**Fig. 9:**
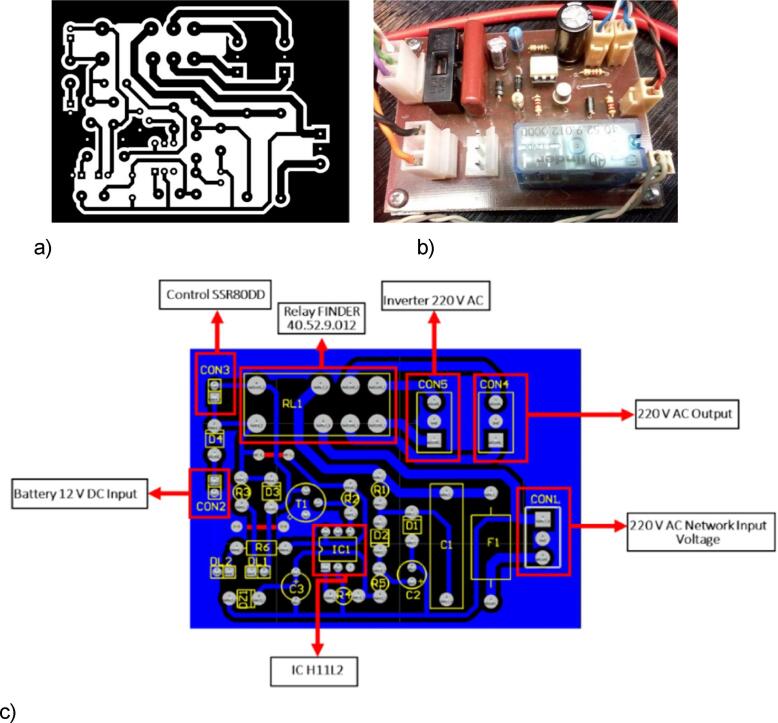
PCB board Tracks (a) Soldered components (b) connection pins(c).

The UPS system (Fig. 8 and Fig. 9) consists of a 500 W inverter with an input voltage of 12 VDC. The DC input voltage to the inverter is controlled by a solid-state relay SSR-80 DD. When the mains power (CON1 input) is not present, a low signal is sent to the IC1H11L2 optocouplerto activates the transistor (T2N2222) to trigger the relay (FINDER40.52.9.012) and connect the 220 VAC output of the inverter to the electrical part of the device.

### 5.5 Arduino control

**Fig. 10:**
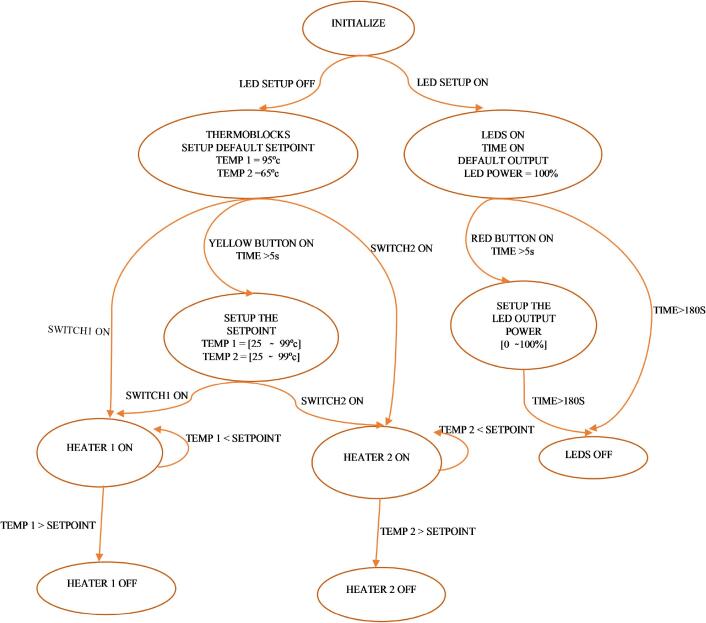
Flow chart state diagram for the Arduino code.

Bang-Bang temperature control with hysteresis (=1°C) is implemented in the distributed Arduino code (Fig. 10). Briefly, the microcontroller reads the current temperature of each thermoblock and switches the heating ON or OFF depending on the result, incorporating a hysteresis to avoid rippling. Firstly, the Thermoblocks are initialized by switching on its power supply system and the user can setup the temperature setpoint (from 25 °C to 99 °C) for each chamber. The default setpoint temperatures for each chamber are Temp1 = 95 °C and Temp2 = 65 °C. The Thermoblocks can be operated simultaneously or independently. If the switch Temp1 or switch Temp2 is ON, the corresponding chamber will start heating. The LCD display always shows the real-time temperature from the chambers. By a click on the temperature setup button, the LCD displays the setpoint temperature values for each Thermoblock.

The developed Arduino code allows the user to regulate the power LED intensity, that can vary from 0 to 100 %. by pressing the input corresponding button. When the microcontroller enters on LED SETUP mode, the LEDs turns ON. During this time, the metal plates of Thermoblocks do not heat up because they are configured to be in passive mode. After 180 s, the LEDs are automatically turned OFF. The user can turn ON again by entering in LED SETUP mode, otherwise, the Thermoblocks SETUP mode operation is started.

The total weight of the assembled device is only 6.5 kg, thus easy to transport.

## 6 Operation instructions

### 6.1 Thermoblocks

The thermoblocks part of the device has simple operation instructions since it was designed to be easy to use and robust for routine work in challenging laboratory environments. After the general power is switched on, the actual temperature of each thermoblock is displayed. The default settings for the thermoblock temperatures are 95 °C and 65 °C, respectively, as indicated by pressing the corresponding button in the front panel. For specific variants of LAMP protocols, the user can modify these temperature settings. To this end, if the button is pressed for more than 5 s, the device enters the ¨SET mode¨, allowing the user to change the values of setpoints of temperatures T1 and T2). In SET mode, the LCD displays “- − − − “ for 3 s, to show that the device is working in SET mode. After 3 s, the current value of Setpoint T1 “65° − − “ appears on the LCD. The user can increase the value of Setpoint T1 up to a maximum of 99° by pressing the button. If the user continues pressing the button, the Temperature value T1 returns to a minimum of 25° it increases until the user stops it by pressing the button (e.g. Set the value to 50° and stop pressing the button). After 5 s with the button on the OFF position, the LCD displays the value of Setpoint T1 on the Left and Setpoint T2 on the Right “50° 95° “. Similarly, by pressing the button, the user can modify the value of Setpoint T2. If the user stops pressing the button for 5 s, the selected values will be stored as the current setpoint for each thermoblock, and at this time, the LCD will display the real measured temperatures of T1 and T2.

After waiting for the period required reaching the target temperature (that the user can check by observing the displayed actual temperature), the device is ready to use. The wells can then be used for placing sample tubes. Each thermoblock is equipped with a thermally isolating lid to help keep wells temperature and minimize heat leaks, thus saving energy. The user can independently switch on/off each thermoblock. It is important that the samples are taken out of the thermoblock (either at 65 °C and 95 °C) at the required time for each process step.

The only obvious safety hazard for the user, which also applies to any equivalent commercially available device, is to avoid touching (bare skin or using conventional lab gloves) the hot thermoblock surfaces (up to 95 °C).

### 6.2 LAMP reaction detection

To observe the fluorescence in the samples after the reaction, the user should place the tubes into the corresponding wells of the viewer chamber (using the piece to protect them from room light), switch on the excitation LEDs by the corresponding button, and look by naked eyes through the viewer chamber orifices.

### 6.3 Setting the LEDs power level

On top of the electronic enclosure is installed a red button which allows the user to adjust the LED intensity value from the default 100 % value to 0 %. If the button is pressed by a short click (time < 5 s), the LCD will display the word ¨*Led¨* and the corresponding power value intensity (e.g. *¨Led 100¨*), then the LEDs will light up for 180 s. After this time, they will turn off automatically. If the button is held down for more than 5 s, it enters SET mode and allows the user to set a new power value output of the LEDs. Pressing the button again increases the power value in 10 % steps (if it is held down it will automatically increase the power every 500 ms). Once the value of 100 % is reached, it will return to 0 % (LEDs off) and subsequently increase gradually. When the user reaches the desired power value, if the button is not pressed for 5 s, the LED power value is stored, and the LCD will display the real-time temperatures T1 and T2 of the Thermoblocks. It should be noted that in SET mode working, the metal plates do not heat up.

## 7 Validation and characterization

### 7.1 Thermoblocks

The first device function is to provide well-controlled temperature wells for many sample tubes by minimizing power consumption. Accordingly, we characterized the setting by carefully measuring temperature and battery consumption under different operating conditions.

Energy consumption was measured in the worst-case scenario, i.e. when a 12 V battery powered the device at low ambient temperatures. Charge consumption was measured by recording the battery current from the voltage drop across a 0.05 Ω (59 W) resistor in series with the battery output. The current signal was integrated to obtain the corresponding charge consumption over time. Simultaneously, the signal of the temperature sensor placed within the aluminum core block was recorded.

**Fig. 11:**
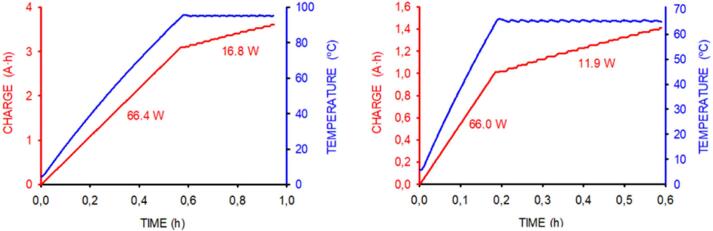
Electric consumption (red) and thermoblock temperature (blue) after switching on the 95 °C (left) and 65 °C (right) thermoblocks. Electric consumption is measured as the charge provided by a conventional 12 V car battery. Numbers in red indicate the measured power consumption. Measurements were carried out at an ambient temperature of 5 °C.

Fig. 11 shows the time course of each thermoblock temperature and charge consumption after switching on the device when the ambient temperature (T_amb_) was 5 °C (to this end, the device was placed within a 5 °C thermostatic chamber). The figure shows that thermoblock temperature almost linearly increased from 5 °C to the target temperature (95 °C or 65 °C). Remarkably, after reaching that temperature, the feedback system did not produce transient oscillations, and the temperature remained steady with oscillations within 1 °C. As indicated in Table 2, the time required to achieve the target temperature from 5 °C was 35.9 min and 10.8 min for the 95 °C and 65 °C thermoblocks, respectively.

**Table 2:** Characterization of the time to reach the steady state target temperature and the electric consumptions after switching on the 95 °C and 65 °C thermoblocks. Data correspond to measurements carried out at ambient temperatures of 5 °C and 23 °C.

	**95 °C** thermoblock			**65 °C** thermoblock		
***Ambient temperature*** ***(°C)***	*Time* *to 95 °C* *(*min*)*	*Consumption to 95 °C* *(A·h)*	*Consumption* *at 95 °C (A·h/h)*	*Time* *to 65 °C* *(*min*)*	*Consumption to 65 °C* *(A·h)*	*Consumption* *at 95 °C (A·h/h)*
**5**	35.9	3.10	1.40	10.8	1.01	0.99
**23**	25.8	2.35	1.19	7.2	0.66	0.75

Battery charge consumption was initially linear (since, below the target temperature, the ≈66 W power was constant and maximal). Table 2 shows that the initial charge consumption required was 3.10 A·h and 1.01 A·h for the 95 °C and 65 °C thermoblocks, respectively. After reaching the target temperature, charge consumption was overall linear (including short on–off power steps as determined by the control system), being 1.40 A·h/h and 0.99 A·h/h for the 95 °C and 65 °C thermoblocks, respectively (Table 2).

Consistently, the measured power during the steady state temperature regime closely corresponds to the expected value from the equation for heat transfer (Q’ = K·A·(T_well_ − T_amb_)/d) through a surface A of thickness d and thermal conductivity K separating different temperatures T_well_ and T_amb_. Indeed, for the thermal isolation box dimensions (16 cm x 16 cm x 14 cm), d = 0.03 m, K=0.04 W·°C ^−1^·m^−1^ for mineral wool, T_well_ = 95 °C, and T_amb_ = 5 °C, heat dissipation Q’ = 16.2 W, which is in keeping with the 16.8 W measured in these conditions (Figure 7.1).

The same previously described measurements were carried out when T_amb_ was 23 °C. The time courses observed were as those in Fig. 11, only changing the times to reach target temperatures and the charge consumption values (Table 2). As expected, at T_amb_ = 23 °C, the initial time to target temperature and initial and steady-state charge consumption were lower than when the device was at T_amb_ = 5 °C.

Data in Fig. 11 and Table 2 indicate that the device provides very well-controlled temperature with an energy consumption allowing considerable continuous operating hours of both thermoblocks with the charge stored in a conventional car battery (e.g., 80 A·h), even when LAMP is applied at T_amb_ = 5 °C. Data on initial and steady-state charge consumptions (Table 7.1) suggests when it is not energetically favorable to maintain the system continuously powered. For instance, at T_amb_ = 5 °C, battery charge can be saved by switching off and subsequently on the 95 °C and 65 °C thermoblocks if no samples have to be processed in the next ≈2.2 h and ≈1h, respectively.

Data in Fig. 11 and Table 2 refer to the excellent performance of the device in terms of its core sensor temperature. However, the individual temperatures at the well are the relevant variables for the user. These were measured by filling all the wells with water to mimic the sample solution inside the tubes and we measured the local temperature with a small, fast-response NTC thermistor (GA10K3MCD1, Measurement Specialties, Galway, Ireland). We found that within all the wells in each thermoblock at its steady state, the temperature was the same with differences < 0.8 °C as compared with the sensor temperature in the aluminum thermoblock core.

**Fig. 12:**
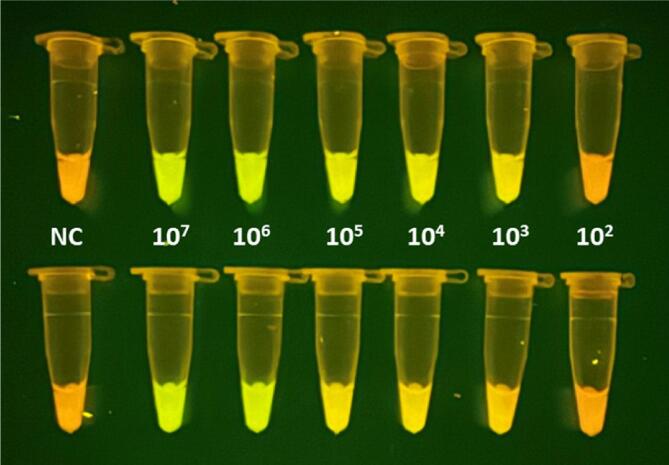
Performance of the open-source thermoblocks (top) compared with that of commercially available laboratory devices (bottom) when LAMP was applied to test *Haemophilus influenzae* samples containing different bacterial concentrations ranging from 1.5x10^7^ CFU/mL to 1.5x10^2^ CFU/mL, including a negative control (NC) with no bacteria. This image is the raw picture obtained by a conventional mobile phone camera in automatic mode.

Fig. 12 shows a practical example of the device performance in a LAMP test on bacterial samples compared to conventional devices. A bacterial suspension with a 0.5-McFarland turbidity standard was prepared in a nephelometer (BD PhoenixSpec, Becton&Dickinson) from a 24-h culture of *Haemophilus influenzae* (ATCC 49766). The concentration of bacteria in this suspension was ≈1.5x10^8^ CFU/mL. Serial 1:10 dilutions were then made to 1.5x10^2^ CFU/mL with a final volume of 900 μL each. These suspensions were brought to 95 °C for 10 min. Subsequently, 10.6 µL was taken from these preparations to be tested for the LAMP reaction at 65 °C for 60 min using SYTO-9 and hydroxy naphthol blue (HNB) as fluorochromes emitting weak green and red fluorescence [18] in samples positive and negative for *Haemophilus influenzae*, respectively. This process was carried out in parallel duplicates, one in the open-source thermoblocks presented here and the other in conventional laboratory devices: TX220 (Analytic Jena) for extraction at 95 °C, and Thermocycler 2720 (Applied Biosystem) for the LAMP reaction at 65 °C. Finally, the processed samples were observed by the naked eye with a blue light (470 nm excitation wavelength) transilluminator (Save Imager 2.0; Invitrogen). Fig. 12 shows a photograph of the processed tubes, showing virtually the same positive (green) and negative (red) detection when processed with the open-source thermoblocks and the conventional laboratory devices.

### 7.2 LAMP reaction detection

**Fig. 13:**
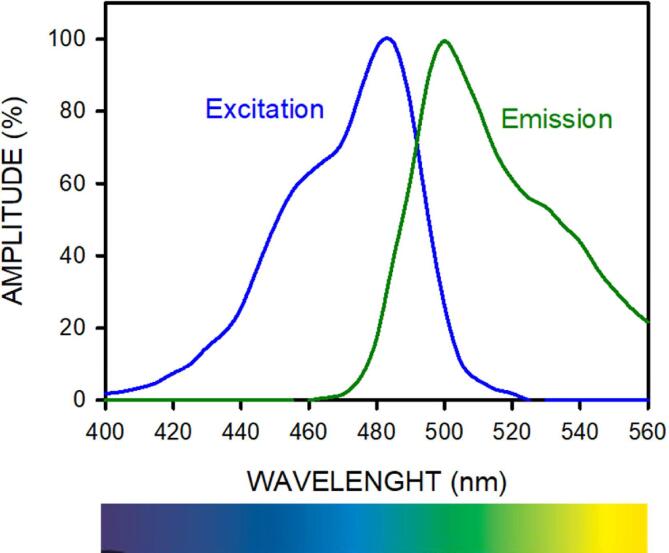
Normalized amplitude of excitation end emission light spectra for fluorochrome SYTO-9.

The second function of the LAMP device is to detect the fluorescence light induced by the positive or negative presence of microbial DNA. The simplest and most robust way for detecting a microorganism in the sample is by naked-eye observation of its color after the LAMP reaction is carried out at 65 °C. Optimal observation depends on whether the light excitation and emission settings in the device are adapted to the fluorochromes used in each application. Specifically, the wavelength spectrum of the excitation light source should be selected to maximally induce fluorescence but lie outside the spectrum of the emitted fluorescence so that the latter can be distinguished. This issue is not critical regarding fluorochrome HNB since excitation occurs for blue wavelengths and emission is in the red wavelengths [18]. As emission and excitations are at both extremes of the visible light spectrum, it is not difficult to find a filter separating them. By contrast, when using fluorochrome SYTO-9, the device requirements are more critical. Indeed, in this case, the spectral bands of excitation and emission are very close, as shown by Fig. 13 (from data obtained in [19]). The excitation bandwidth is mainly placed in the blue section of the light spectrum (maximum at 483 nm), and emission is around the adjacent green-yellow wavelengths (with maximum at 500 nm), thus being critical in how excitation and emission lights are distinguishable.

**Fig. 14:**
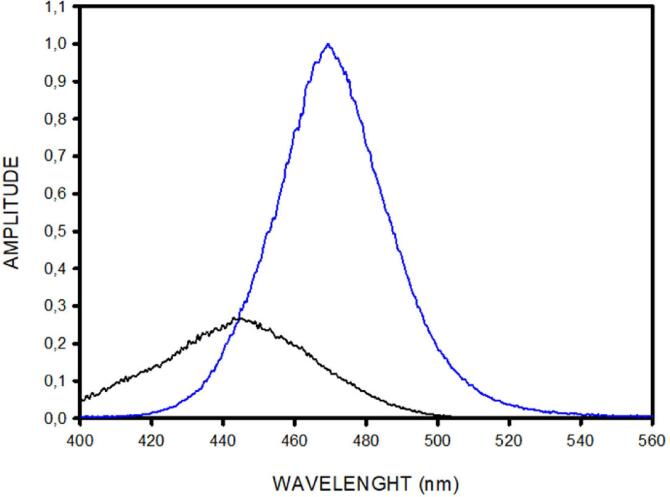
Low-cost excitation setting for light excitation in the open-source device described herein. Blue line is the normalized emission spectrum of the blue LED, and black line is the transfer function of the blue screen filtering the light emitted by the LED.

**Fig. 15:**
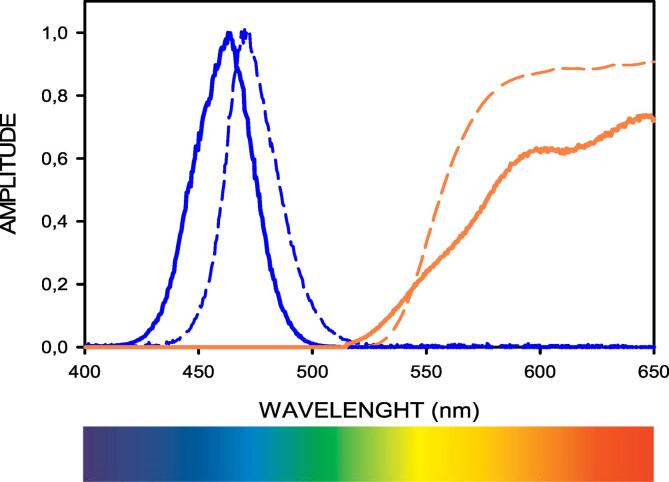
Normalized emission spectrum (blue) and transfer function of the observation filter (amber). Solid lines correspond to the open-source device described herein. Dashed lines correspond to the conventional Safe Imager 2.0 Blue Light Transilluminator (ThermoFisher).

The low-cost excitation setting in our open-source device was optimized by selecting the LED and the filters used for tailoring the excitation light and the detection filters. All optical characterizations (light spectra and filter transfer functions) were measured with a spectrophotometer (HR2000CG-UV-NIR, Ocean Optics). The emission spectrum of the blue LED and the transfer function of the blue filter (079-Tokyo Blue, LEE Filters) to modulate LED light are shown in Fig. 14. The resulting excitation spectrum applied by our device to the samples is shown in Fig. 15 (blue solid line). Therefore, the excitation light lies within the SYTO-9 absorption spectrum but is restricted to wavelengths below 500 nm, thus not invading the band of maximal green emission (Fig. 13).

**Fig. 16:**
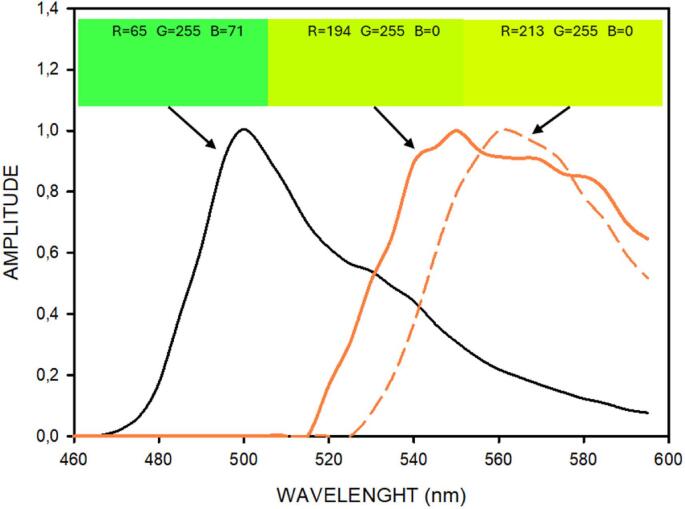
Normalized emission spectrum of fluorophore SYTO-9. Black line: fluorescence emission spectrum (as in Fig. 13). Thick solid amber line: simulated spectrum resulting by filtering the emission spectrum with the screen used in this open-source device. Thin dashed amber line: same for the screen of the conventional Safe Imager 2.0 Blue Light Transilluminator (ThermoFisher). The filter transfer functions employed are shown in Fig. 15 (same line formats). The color, and its RGB components, corresponding to each of the spectra are shown in the inserts.

The screen filter to observe the emission fluorescence was carefully selected by analyzing the transfer function of a series of low-cost color screens (e.g., filters 015, 105, 158, 179, 204 from LEE Filters). Fig. 15 shows the transfer function of the filter implemented, which consists of 4 commercially available screens (015-Deep Straw, LEE Filters) placed in series. It should be mentioned that the transfer function of 4 identical screens in series (H_4_(λ)) is the 4rt power of the transfer function of one individual screen (H_1_(λ)), i.e., H_4_(λ) = (H_1_(λ))^4^. Interestingly, the wavelength for which the excitation light spectrum vanishes (≈525 nm) virtually coincides with the one for which the transfer function allows light transmission. In this way, the excitation light can be eliminated while a substantial part of the green components of emission (Fig. 13) can be observed. For the sake of comparison, Fig. 15 also shows the transfer function of the observation screen in the conventional Safe Imager 2.0 Blue Light Transilluminator (ThermoFisher), showing that this device is slightly less optimal for allowing observation of relevant green components (525–540 nm) in emission light. This is illustrated by a simulation in Fig. 16, showing the emission spectrum of SYTO-9 (as in Fig. 13), and the result of multiplying this spectrum for the transfer functions of the screen selected for this open-source device and the one of the screen in the previously mentioned common commercial device. The figure clearly shows our device optimizes observation of the emission green components in the relevant ≈515-555 nm wavelengths band, as realized by the color appearance of the corresponding lights. This color simulation was made by adding the RGB components of each wavelength [20] multiplied by the corresponding amplitudes of the spectra in Figure 7.6. It is apparent in the figure that the green color from our screen approaches the original emission color more closely than the one from the commercial device.

**Fig. 17:**
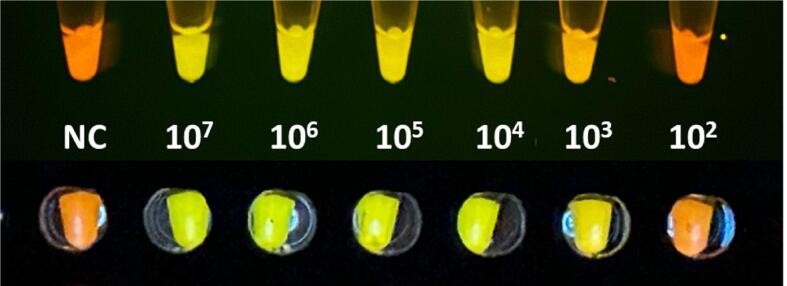
Comparison of the fluorescence observed by the open-source viewer (Bottom) and the fluorescence observed with a conventional device (Top) when LAMP was applied to test the same tubes with *Haemophilus influenzae* samples containing different bacterial concentrations ranging from 1.5x10^7^ CFU/mL to 1.5x10^2^ CFU/mL, including a negative control (NC) with no bacteria. This image contains the raw pictures obtained by the same conventional mobile phone camera in automatic mode.

As an example of the excellent performance of the low-cost fluorescence observation setting in the open-source device (LED, filters and viewer chamber), Fig. 17 shows the same LAMP sample tubes as also observed with a commercial transilluminator (Safe Imager 2.0 Blue Light Transilluminator, ThermoFisher). In agreement with Fig. 16, the open-source setting described herein shows the green (positive) component more clearly than the commercial device.

## CRediT authorship contribution statement

**Jorge Otero:** Writing – review & editing, Writing – original draft, Methodology, Conceptualization. **Miguel A. Rodríguez-Lázaro:** Methodology, Validation. **Arturo Martínez-Trejo:** Methodology, Conceptualization. **Daniel Mbanze:** Writing – original draft, Methodology. **Gorka Solana:** Methodology. **Andrea Vergara:** Investigation. **Salvador Bosch:** Methodology. **David Gozal:** Investigation. **Jordi Vila:** Funding acquisition, Conceptualization. **Ramon Farré:** Writing – review & editing, Writing – original draft, Supervision, Methodology, Funding acquisition, Conceptualization.

## Declaration of competing interest

The authors declare that they have no known competing financial interests or personal relationships that could have appeared to influence the work reported in this paper.
